# “I don’t know what I’m feeling for”: young women’s beliefs about breast cancer risk and experiences of breast awareness

**DOI:** 10.1186/s12905-023-02441-w

**Published:** 2023-06-16

**Authors:** Sarah Hindmarch, Louise Gorman, Rhiannon E. Hawkes, Sacha J. Howell, David P. French

**Affiliations:** 1 Manchester Centre for Health Psychology, Division of Psychology and Mental Health, School of Health Sciences, Faculty of Biology, Medicine and Health, University of Manchester, Manchester, UK; 2 NIHR Greater Manchester Patient Safety Translational Research Centre, University of Manchester, Manchester Academic Health Science Centre, Manchester, UK; 3 Division of Cancer Sciences, Faculty of Biology, Medicine and Health, University of Manchester, Manchester Academic Health Science Centre, Manchester, UK

**Keywords:** Breast cancer, Breast awareness, Breast self-examination, Risk perceptions, Qualitative research

## Abstract

**Background:**

Younger women are often diagnosed with advanced breast cancer. Beliefs about risk are instrumental in motivating many health protective behaviours, but there may be confusion around which behaviour is appropriate to detect breast cancer earlier. Breast awareness, defined as an understanding of how the breasts look and feel so changes can be identified early, is widely recommended. In contrast, breast self-examination involves palpation using a specified method. We aimed to investigate young women’s beliefs about their risk and experiences of breast awareness.

**Methods:**

Thirty-seven women aged 30–39 years residing in a North West region of England with no family or personal history of breast cancer participated in seven focus groups (n = 29) and eight individual interviews. Data were analysed using reflexive thematic analysis.

**Results:**

Three themes were generated. *“Future me’s problem”* describes why women perceive breast cancer as an older woman’s disease. *Uncertainty regarding checking behaviours* highlights how confusion about self-checking behaviour advice has resulted in women infrequently performing breast checks. *Campaigns as a missed opportunity* highlights the potential negative effects of current breast cancer fundraising campaigns and the perceived absence of educational campaigning about breast cancer for this demographic.

**Conclusions:**

Young women expressed low perceived susceptibility to developing breast cancer in the near future. Women did not know what breast self-checking behaviours they should be performing and expressed a lack of confidence in how to perform a breast check appropriately due to limited knowledge about what to look and feel for. Consequently, women reported disengagement with breast awareness. Defining and clearly communicating the best strategy for breast awareness and establishing whether it is beneficial or not are essential next steps.

**Supplementary Information:**

The online version contains supplementary material available at 10.1186/s12905-023-02441-w.

## Background

Epidemiological studies from the past decade have illustrated the growing burden of breast cancer in pre-menopausal women worldwide [1–4]. Compared to post-menopausal women, younger women are more likely to develop unfavourable breast cancer subtypes, which are associated with higher recurrence and mortality despite aggressive treatment regimens [5, 6]. Additionally, young women often present at an advanced stage or have a delayed diagnosis because of a low index of suspicion by the patient and primary doctor [1, 7]. Consequently, enhancing recognition of symptomatic presentation amongst this group is likely to result in earlier help-seeking behaviour and thus earlier stage diagnosis [8].

Recommendations for what behaviours women should be performing to facilitate early detection of breast cancer has changed over time. From 1950, teaching breast self-examination (BSE) to women by healthcare professionals was recommended. BSE is the palpation of a woman’s breasts for self-detection of breast cancer at a specific time each month according to a rigorous set method [9]. However, in 2003, a Cochrane review demonstrated that regular BSE does not result in a reduction in breast cancer mortality [10]. The same review also showed that potential harms including unnecessary biopsies and health anxiety were increased in comparison with control groups [10]. This led to the abandonment of routinely teaching women BSE as a recommended practice for healthcare professionals in the UK and USA [11, 12], and the removal of BSE from clinical recommendations [13]. However, most breast cancers in younger women are detected after the development of symptoms. In one US study, 71% of cases of breast cancer in women younger than 45 years were detected by the women themselves [14]. In recent years, ‘breast awareness’ has replaced BSE and has been strongly promoted by breast cancer charities and health authorities. Breast awareness involves individuals knowing what is normal for them and the signs and symptoms of breast cancer so that any concerning changes can be acted upon [15]. Breast awareness should not include recommendations for regular implementation of a set method for breast checking.

Healthcare professionals have expressed concerns that the distinction between breast awareness and BSE is unclear, with references to both terms in the same guideline documents potentially causing confusion for both healthcare professionals and women [16]. There is evidence supporting this confusion amongst healthcare professionals; 50% of US obstetrician-gynaecologists surveyed in one study did not know there were recommendations against routine BSE in national guidelines [17]. However, whether confusion is present amongst young women regarding which breast self-checking behaviours they should be performing remains unknown. There is a dearth of qualitative studies, conducted since the recommendations changed, examining pre-menopausal women’s views and experiences of breast awareness [18].

Breast awareness recommendations indicate women should be engaged in self-checking behaviour [15]. To understand whether women engage in these behaviours, it is useful to consider women’s perceptions of risk. The presence of family history has been found to dominate women’s breast cancer risk perceptions, with other indicators of risk such as breast density typically ignored [19, 20]. Previous research has demonstrated that risk perceptions are a key predictor of many health protective behaviours including breast screening attendance [21, 22]. Given that breast awareness is a health protective behaviour and previous research has been limited to screening age and high-risk populations, it is important to explore younger women’s beliefs about breast cancer risk.

The present analysis reports on data collected from a study which had the primary aim of investigating women’s views on, and requirements for, the delivery of breast cancer risk assessment [23]. However, during the course of data collection for this study, a large volume of unanticipated data was elicited regarding young women’s beliefs about their own breast cancer risk and their experiences of breast awareness. The aim of the present analysis is therefore to examine young women’s beliefs about breast cancer risk and experiences of breast awareness.

Specific objectives were to:

- Explore women’s understanding of breast cancer risk.
- Identify the factors contributing to women’s beliefs about their own breast cancer risk.
- Explore women’s understanding and experiences of breast awareness.

## Methods

### Design

A cross-sectional qualitative design was used. As the topic of risk assessment and screening was theoretical to participants, focus groups were deemed the most appropriate method of facilitating discussion. Focus groups allow for reflection and clarification of perspectives, adding depth to the data [24]. They also allow for perspectives to evolve and become co-created during the discussion, allowing insight into the degree of group consensus on the topic [25]. Where participants were unable to attend scheduled focus groups, individual interviews were carried out instead.

### **Participants and setting**

Participants were recruited through responding to study advertisements outlining the topic and inclusion criteria. Participants were eligible if they were: [1] born female, [2] aged 30–39 years, [3] residing in Greater Manchester in England, [4] able to provide informed consent, and [5] able to understand and communicate in English. Women who had received a breast cancer diagnosis or had a first-degree female relative (mother or sister) affected by breast cancer were not eligible to participate.

### Procedure

Advertising posters were shared on Facebook and Twitter and were also displayed on noticeboards in three local libraries and community centres. After seeing an advertisement on Twitter, the co-founder of a local ethnic minority organisation made direct contact with the research team and facilitated the recruitment of four members of their community. Prospective participants were screened against inclusion criteria via email/telephone and sent participant information sheets.

The topic guide was developed to address the primary aim of the study, which was to investigate young women’s views on, and requirements for, the delivery of breast cancer risk assessment, and informed by a review of the literature (see Additional file 1). The lead author developed an initial draft and this was reviewed by public contributors and the rest of the research team who have expertise in medical oncology, breast cancer and screening services, health services research, health psychology and qualitative methods. The content and structure of the topic guide was revised in line with the feedback received. Data were collected between February and November 2020, at first in person then later via telephone and Zoom conferencing due to COVID-19 restrictions. Participants gave demographic information (age, ethnicity and home address postcode) prior to data collection. The full residential postcode of participants was used to extract the Index of Multiple Deprivation decile, a measure of relative deprivation for small areas in England, with 1 representing the most deprived 10% of small areas in England and 10 the least [26]. Data were audio-recorded and transcribed verbatim. A recording malfunction occurred during one online focus group meaning that only the first half of discussion was recorded. Field notes were taken to capture insights from the remaining discussion. Identifiable information was anonymised, and participants were assigned pseudonyms. Focus groups were facilitated by two female researchers (SH and REH) with postgraduate qualitative research training and experience. Interviews were conducted by the lead author (SH). Data collection continued until the research team were satisfied that sufficient data had been collected to answer the research question [27]. Participants were compensated for their time with a £20 cash payment.

### Patient and public involvement

Public contributors were involved in the design of this study. A research advisory group, consisting of five minority ethnic community leaders, advised on the research idea and intended recruitment procedures. Wording and terminology used in the recruitment poster and participant information sheet was revised following feedback from nine Black African women aged 30–39 years. During topic guide development, four White women aged 30–39 years advised on wording of questions, prompts and flow.

### Researcher positioning

The lead author (SH) was a White British female doctoral researcher with a background in Psychology. She has worked in cancer prevention and early detection research for six years, both reflecting and shaping her positive views of early cancer detection initiatives. SH is 31 years old meaning she was cognisant of the participant’s life stage and had life experiences in common with some of her participants, for example cervical screening. Other members of the research team were a breast cancer clinician working in cancer prevention and early detection and experienced academics who have reservations about the extent to which prevention and early cancer detection initiatives allow for informed choices about participation. The data resonated with feminist views of the team meaning that data analysis and interpretation were informed by a desire to legitimise and honour women’s absent or silenced experiences.

### Data analysis

The present analysis included responses to questions focused on comprehension of breast awareness, confidence in being breast aware, and awareness and thoughts about the current breast cancer incidence and mortality rates in younger women (see Additional file 1). This data was coded during the initial coding of the complete dataset but it did not form part of the framework for answering the primary aim of the study which was to understand women’s views on, and requirements for, the delivery of breast cancer risk assessment [23]. The inductive analysis reported in this paper focuses on the new data generated by the focus groups and interviews as pertinent to understanding how and why women hold particular opinions on breast cancer risk and breast awareness.

Data were analysed using reflexive thematic analysis as this allows the flexibility to combine multiple sources of data and is suited to research examining views about particular phenomena [28, 29]. A critical realist approach was taken meaning we treated the data as indicating the participants’ perception of their reality, which is shaped by and embedded within their cultural context and language [30]. Primary data analysis was conducted by the lead author with input from LG and DPF. The lead author familiarised themselves with the focus group and interview data by listening to the audio-recordings and reading the transcripts multiple times. An inductive approach was taken to analysis, working with the data from the bottom-up, to align with our interest in the experiences and perspectives of the participants. Line-by-line coding was conducted by the lead author using NVivo12. The majority of coding was semantic, capturing explicitly expressed meaning and staying close to the language of participants. However, there was a shift towards more latent coding as the analysis progressed. Related codes were then grouped together to form broader patterns of meaning and a set of initial themes were developed using thematic mapping. The initial themes were evaluated by reviewing the coded data extracts and the entire dataset to ensure the analysis was grounded in the data. The final analysis was the result of multiple rounds of theme refinement through writing (by the lead author) and discussion between members of the research team (SH, LG and DPF).

Important techniques for ensuring quality reflexive thematic analysis are the researcher’s depth of engagement with the data and reflexive practice, rather than measures of accuracy or reliability [31]. The lead author kept a research journal throughout the research process to allow ongoing reflection about how her prior knowledge and assumptions were influencing data collection and analysis and guiding decision-making. Meetings with LG and DPF during the data analysis phase of the research provided opportunities for the lead author to explain and clarify thinking and further reflect on how the assumptions she brought to data analysis might be delimiting her data engagement and interpretation.

## Results

Thirty-seven women took part; seven focus groups (participant range n = 3–5) and eight interviews were conducted. Focus groups ranged from 71 to 90 min (median 85 min) with five conducted via Zoom conferencing and two face-to-face. Interviews lasted between 40 and 81 min (median 55 min) with five conducted by telephone and three face-to-face. Most participants were White British (see Table 1). Deprivation deciles ranged from 1 to 10, with a median decile of 4 indicating that a substantial proportion of the sample were recruited from more deprived areas.

**Table 1:** Sample demographics (n = 37)

Characteristic	N (%)
Age range (years)	
30–33	18 (49)
34–36	7 (19)
37–39	12 (32)
Ethnicity	
White British	29 (78)
Black African	4 (10)
White (Other)	1 (3)
Indian	1 (3)
Mixed (White/Arab)	1 (3)
Mixed (English Caribbean)	1 (3)
Level of deprivation^a^	
Low (8–10)	4 (10)
Medium (4–7)	18 (49)
High (1–3)	15 (41)

^a^Ranked in deciles according to the Index of Multiple Deprivation 2019, a measure of relative deprivation for small areas in England (Ministry of Housing, Communities & Local Government, 2019).

Three themes were generated from the data: [1] “Future me’s problem”, [2] Uncertainty regarding checking behaviours, and [3] Campaigns as a missed opportunity. Quotes are presented with a pseudonym followed by interview (I) or focus group (FG) number.

### Theme 1: “Future me’s problem”

Participants conceptualised breast cancer as an ‘older woman’s disease’. Many participants said they did not perceive breast cancer as an immediate health concern, for example one participant stated it was *future me’s problem (Florence, FG5).* Women attributed this perception to the organisation of the NHS breast screening programme which invites women aged 50–70 + years. Participants reported that given finite NHS resources, they assumed that breast screening is offered when the likelihood of developing breast cancer is greatest. The absence of a breast screening programme for younger women and lack of communication from healthcare professionals regarding breast awareness was perceived to indicate lesser risk for this age group, as women expected to be told by health authorities if they were at risk of developing a disease. Consequently, women expressed low perceived susceptibility to developing breast cancer.

*I thought the risk went up after 50 and that’s the whole reason that we have the screening after 50 (Debbie, FG5)*.

*I think we believe innately that if we’re at medical risk, we’ll just be told about it. Yeah, or we would expect to hear from the doctors about it or, you know, it would be more present in our minds, yeah. (Laura, I1)*

Some women considered healthcare interactions like cervical screening and antenatal appointments as missed opportunities to discuss breast awareness and whether breast cancer was a relevant health concern for their age group. In line with this, women reported accessing limited, if any, information about breast health.

Breast cancer as an ‘older woman’s disease’ has been reinforced further when women reported consulting healthcare professionals with breast health concerns or concerns about risk. Some women reported feeling embarrassed about seeking help because they feared wasting NHS time. Women described feeling like a burden throughout the care pathway, in both primary and secondary care interactions. Participants who spoke about their experiences of seeking help reported an absence of reassurance following clinical interactions. They reported feeling as though their concerns had been dismissed in Primary Care because of their age.

*I went to a doctor once, I was about twenty-two, and said, “Oh, I’ve got weird lumps in my breasts” And she went, “No, that’s just water retention” and was really dismissive […] I just think that it would be better for doctors not to turn around and tell you you’re far too young when you have a genuine concern. (Nancy, FG2)*

*I’ve got breast cancer on my dad’s side. And I asked the GP what – whether that meant anything. And he was really like, “Whatever. You know, don’t waste my time,” kind of thing. (Carrie, FG2)*

Women who had experienced being referred to secondary care reported feeling reassured after undergoing further investigations and receiving the all clear but conversely this was also seen to confirm their fears of having wasted NHS time and resources. One woman told us that a secondary care healthcare professional’s demeanour during their clinical interaction left her feeling as though she had been referred unnecessarily for a biopsy, *“the guy who did the biopsy was like, almost seemed annoyed that I’d been sent there” (Polly, FG1)*. Women reported that these types of experiences might have a negative impact on future help-seeking behaviour:

*the thought of if I did find something again, went again, again there was nothing there, that feeling of again I’ve, sort of, wasted a bit of NHS time, not that anyone ever made me feel like that but I suppose I used to think, you know, how many times can you do this. (Jodie, FG5)*

When considering cultural differences in breast cancer prevalence and screening, some Black African participants expressed the view that breast cancer was more prevalent in the UK in comparison to their home countries in Africa. This was attributed to differences in diet and environment. Furthermore, these participants described very few instances of and conversations about breast cancer in their communities, which may adversely affect performance of breast self-checking behaviours.

*when I was back home anyway growing up there, cancer was not a prevalent disease […] it wasn’t something that was common as it is over here so when people move over here […] you feel like cancer’s not a thing that affects people of your kind. (Rebecca, FG6)*

### Theme 2: Uncertainty regarding checking behaviours

When asked for their understanding of being breast aware, women believed they should be regularly checking their breasts to know how they normally look and feel so any changes that are not normal for the woman can be detected and reported to a GP. For one participant, being breast aware did not hold any meaning, *I’d say that term doesn’t really mean much to me (Zoe, FG3)*. Some women believed breast checks should be performed monthly and that a specific technique should be used, resulting in the perception there was a ‘correct’ method of checking. Contrary to these beliefs, participants reported infrequently performing breast checks and being unaware of the recommended frequency for enacting this behaviour. Women tended to engage in this behaviour only when prompted by a trigger such as hearing about a relative or peer’s breast cancer diagnosis or media coverage about breast cancer. Different reasons were reported for infrequent engagement with breast checking behaviour. For some women, the anxiety and fear of potentially detecting breast cancer was a barrier to performing the behaviour:

*you still have that anxiety don’t you of “oh gosh, could it be?” and I sort of notice within myself that I feel reluctance to check because of that which I know is silly. (Jodie, FG5)*

Most participants reported not routinely engaging in breast checking due to a lack of confidence in how to perform the behaviour and limited knowledge about what to look and feel for. More specifically, participants expressed difficulty in distinguishing between a concerning and normal change, given natural variation during a menstrual cycle and the impact of breastfeeding. In addition, women mentioned the individuality of breasts, in terms of differences in size and consistency, rendering video demonstrations somewhat ineffective.

*you already know that you should do it you just don’t know what to do. (Joyce, FG1)*

*I’m breastfeeding at the moment so my boobs change on a daily basis […] at the moment, I’d have no idea if something was to do with that or if it was to do with something more, kind of, nasty I suppose. (Brooke, FG3)*

*I don’t know what I’m feeling for, I’ve watched a YouTube video, I’ve watched it and thought, okay, my boobs don’t look like her boobs, but she’s doing all this […]. It didn’t help me. (Miranda, FG1)*

Taken together, these comments highlight that women are finding it difficult to identify their own baseline normal, the reference point needed to make decisions about which changes require action. As a result, training on how to check and education about normal changes was desired in order to increase confidence.

### Theme 3: Campaigns as a missed opportunity

Breast cancer was perceived to have a high profile in the media. Women were familiar with fundraising campaigns such as Race for Life [32] and Wear It Pink [33]. These campaigns were perceived to have served a purpose in raising awareness of breast cancer and destigmatising the disease. However, women felt these campaigns had contributed to the depiction of breast cancer as *‘pink and pretty’ (Natasha, FG4)* in comparison with other cancers. Some believed this portrayal had the inadvertent consequence of lessening the seriousness of breast cancer:

*I think it’s interesting around how breast cancer is portrayed as well, it’s given a bit of a different thing like then say lung cancer or bowel cancer. It does seem a bit pink and fluffier and therefore a bit less scary in a way so therefore people don’t take it as seriously (Gemma, FG4)*.

*It [breast cancer] feels like it’s the less serious of the cancers if that makes any sense [laughs]. It’s like you’ve got your cervical cancer and your bowel cancer and your prostate cancer, and breast cancer is somewhere down the list. (Hannah, I5)*

In contrast to fundraising campaigns, women reported limited exposure to educational campaigns aimed at their age group for raising awareness of breast cancer symptoms, risk-reducing measures and preventive strategies. This was evidenced further by a lack of knowledge about preventive measures. Many women were familiar with CoppaFeel! [34], a breast health awareness charity, but it was viewed as targeting a younger demographic. Given the recent focus of public health campaigning for this age group on attending cervical screening [35], some women perceived cervical cancer as their most serious health risk.

*you get like breast cancer awareness month but besides people wearing ribbons I don’t know really what that’s saying to the world, it’s just oh breast cancer exists which we all know but we don’t know how to avoid that or what we can do about that, so there’s nothing, as far as I’m aware, there’s nothing preventative that’s being put out there (Zoe, FG3)*.

When asked what an effective campaign would look like, women expressed a desire for campaigns focused on effecting behaviour changes relevant to breast health such as advice to reduce risk and checking breasts to increase help-seeking behaviour. Women recommended that campaigns should be delivered in line with their target audience’s preferred means of interaction. For this reason, social media was regarded as the platform of choice for delivery for women in this age group. Several women recalled having seen the ‘Cervical Screening Saves Lives’ national campaign from 2019 which included significant amounts of social media advertising. In addition, women thought it would be important to have a ‘face’ for any campaign in the form of a relevant celebrity or influencer who their age group could identify with and relate to in order to increase the likelihood of paying attention. Instagram was considered a particularly favourable platform because it would lend itself to more engaging visual content such as videos. Some women thought breast awareness should be taught in schools alongside sex education to form a habit of being breast aware from a younger age. The perceived benefit of this was to help normalise and encourage discussions about breast health with peers so it would become part of mainstream conversation.

*you have sex education why aren’t you having some sort of like body education like here is how you check yourself (Zoe, FG3)*.

## Discussion

### Summary of main findings

Participants perceived breast cancer as a distant future health concern and expressed low perceived susceptibility to developing the disease because of the focus of breast screening at 50 years and above. This perception has been reinforced further by women’s experiences of help-seeking whereby they felt their breast health concerns have been dismissed because of their age. Uncertainty about what women should be doing with respect to breast health was apparent resulting in infrequent reports of performing breast checks and hesitancy towards seeking help for breast health concerns. Women described the potential negative effects of current breast cancer fundraising campaigns in terms of lessening perceptions of the seriousness and severity of breast cancer and perceived absence of educational campaigning about breast cancer targeted at their age group.

### Relevance to existing literature

In the present study, women expressed low perceived susceptibility to developing breast cancer in the near future. Although women aged 30–39 years are at a lower absolute risk of developing breast cancer than older age groups, breast cancer is more frequently fatal in younger women than in those diagnosed aged over 50 years [36]. As a result, breast cancer is the leading cause of death in women aged 35–49 years in the UK with 2,000 deaths reported per year [37]. Identifying young women at increased risk of developing breast cancer would allow them to receive the benefits of earlier screening and preventive strategies. A recent review determined that breast cancer risk assessment for women under 50 years currently satisfies many of the standard principles for screening [38]. The feasibility of offering breast cancer risk assessment to women aged 30–39 years is currently being investigated [39].

Risk perception has been found to influence the symptom interpretation process of help-seeking behaviour with those expressing low perceived susceptibility to breast cancer delaying help-seeking [40]. Furthermore, previous research has demonstrated that dismissive interactions with general practitioners induce a worry of unnecessary help-seeking [41]. The findings of the present study suggest that these interactions could also reduce future help-seeking behaviour. This is concerning as women who delay seeking help for symptoms of breast cancer have a reduced chance of survival [42].

Women reported more exposure to fundraising campaigns compared to educational campaigns. This is consistent with breast cancer awareness messaging in recent years, which has shifted from a focus on diagnosis and prevention to fundraising efforts [43]. For this age group, the preferred mode of delivery for campaigns was social media and women recommended partnering with relatable celebrities or influencers as spokespersons. Previous research has demonstrated that celebrities increase the reach of messages on social media platforms such as Twitter in comparison with individuals and organisations [44]. Women desired campaigns that convey actionable breast health messages such as how to perform a breast check. Examination of social media campaigns during breast cancer awareness month reveal that messaging primarily focuses on awareness and support rather than actionable health messages, suggesting that minimal behaviour change will occur as a result [44–47]. The findings discussed here suggest that current breast cancer campaigning is ineffective and does not meet the needs of women aged 30 to 39 years.

Women reported infrequently performing breast checks despite believing they should be enacting the behaviour. In this study, lack of confidence in how to perform a breast check, distinguishing between normal and concerning changes, limited knowledge about what to look and feel for and fear of potentially detecting breast cancer were identified as contributing factors to women’s disengagement with breast checking behaviours. Additionally, aspects of BSE were evident in women’s understanding of breast awareness such as believing there was a recommended frequency to perform breast checks and a ‘correct’ method of checking. Inaccurate understandings of current recommendations for self-checking behaviour have also been found amongst women older than 50 years, whereby women explicitly cited engaging in BSE and only alluded to engaging in breast awareness [48]. These findings are in line with concerns previously raised by healthcare professionals that the distinction between breast awareness and BSE lacks clarity [9, 16]. Currently, breast cancer charities are promoting a variety of breast awareness recommendations to women in the UK, which make it difficult to separate the two concepts. For example, CoppaFeel! offers monthly text message reminders to prompt women to check their breasts (in line with breast self-examination) whilst also providing a self-checkout tool which states there are no rules for checking (in line with breast awareness) [34]. It has been argued that breast awareness is a euphemism for BSE and places an “undue burden” on women to maintain the responsibility to detect their own breast cancer, despite evidence of harms outweighing the benefits [16, 49]. Therefore, it is apparent that clarity is needed about what behaviours are recommended and how this should be communicated to address the confusion women are experiencing.

### Strengths and limitations

The data analysed were responses to the broad introductory questions about breast awareness that were asked to ease participants into conversation before exploring the topic of primary interest which was the delivery of breast cancer risk assessment. Therefore, we did not ask any direct questions regarding beliefs about breast cancer risk. Nevertheless, women spontaneously chose to discuss this indicating it was an important topic to them and further probing of initial responses was conducted to obtain more in-depth information.

Ethnic minority groups and those from a low socio-economic background are underrepresented in cancer prevention and early detection research despite being disproportionately affected by cancer [50]. Therefore, the sample diversity in terms of ethnic minority representation and socio-economic status is regarded as a strength of the present study. However, we acknowledge that the views of other minority groups were underrepresented.

### Implications and future research directions

This research has demonstrated that women aged 30–39 years report uncertainty about what they should be doing with respect to breast health and a lack of confidence in how to perform a breast check appropriately due to limited knowledge about what to look and feel for. Consequently, women reported disengagement with breast awareness. To what extent this disengagement is concerning remains unknown as breast awareness continues to be promoted without any evidence of benefit. In the absence of clear evidence of benefit, future research should attempt to define the best strategy, in terms of recommended behaviours, for breast awareness. This could facilitate consistent messaging so women know what they should be doing.

In the meantime, breast cancer charities should consider delivering and evaluating an educational campaign targeted at this demographic which clearly specifies which breast changes to be concerned about if identified. This is particularly important given current interest in offering breast cancer risk assessment to young women which will be highlighting the importance of remaining breast aware and performing breast checks in risk feedback. Further research with minority and marginalised groups including transgender individuals should be conducted to inform the design of future campaigns, as they are likely to have different needs and therefore recommendations for breast awareness communication [51]. The role of technology in assisting women to perform breast self-checks should be considered given the current development of Dotplot, an at-home breast health monitoring tool offering guided self-checks on a monthly basis to enable early detection of changes in breast tissue composition that could be cancer [52].

The present study also highlighted that young women do not perceive breast cancer as a relevant and immediate health concern. This perception has been reinforced by clinical interactions with healthcare professionals who were dismissive about breast health concerns. Therefore, future qualitative research should examine primary healthcare professionals’ understanding of breast cancer risk and referral decision-making for young women to inform the development and evaluation of educational interventions aimed at improving consultations about breast health concerns amongst this age group.

## Conclusions

Women aged 30–39 years perceived breast cancer as a future health concern. They reported not knowing what breast self-checking behaviours they should be performing and expressed a lack of confidence in how to perform a breast check appropriately due to limited knowledge about what to look and feel for. Consequently, women reported disengagement with breast awareness. Defining and clearly communicating the best strategy for breast awareness and establishing whether it is beneficial or not are essential next steps.

## Electronic supplementary material

Below is the link to the electronic supplementary material.

Additional file 1: Focus group and interview topic guide.

## Data Availability

The data that support the findings of this study are available from the corresponding author, SH, upon reasonable request.
