# Supplementary material for: “I don’t know what I’m feeling for”: young women’s beliefs about breast cancer risk and experiences of breast awareness
Source: BMC Womens Health. 2023 Jun 16;23:312. doi: 10.1186/s12905-023-02441-w (PMC10276361; doi:10.1186/s12905-023-02441-w)
Supplement: Supplementary file 1 — Additional file 1: Focus group and interview topic guide. [file 12905_2023_2441_MOESM1_ESM.docx]

**Additional file 1. Focus group and interview topic guide**

**Breast awareness/behaviours**

1. What is your understanding of being breast aware?

- Probe: Can you tell me more about that?

1. How aware and well informed do you think women in your age group are about being breast aware?

- Prompt: How aware do you think women in your age group are of the current statistics of breast cancer? Remind of stats if needed
- Probe: Can you tell me more about that?

1. How confident are you in being breast aware?

- Prompt: What information sources, if any, do you look at for breast health?
- Prompt: Awareness of what to look for, confidence in carrying out self-examination (behaviours)
- Probe: What are your reasons for feeling that way?

**Acceptability of risk and process of risk assessment**

As stated earlier, future breast cancer risk can be measured by completing a risk assessment which combines results from three pieces of information. These three things are a questionnaire, a spit sample and a mammogram (breast x-ray).

1. What’s your initial reaction to offering women the opportunity to find out their future breast cancer risk?

- Prompts: Benefits, concerns, questions
- Probe: Can you tell me more about that?

1. How would finding out this information make you feel?

- Probe: What are your reasons for feeling that way?

1. To find out risk, women would need to answer questions about family history of breast & ovarian cancer, hormonal factors such as age at first period and personal information such as weight and number of pregnancies.

- How would you feel about answering these sorts of questions?

Probe: What are your reasons for feeling that way?

Prompt: Mention the different categories of questions if not addressed

- What could stop women completing the questions?

Prompt: How could these barriers be minimised?

- What could be done to help women complete the questions?

Prompt: What would be your preferred route (online, telephone etc) for accessing help?

1. To find out risk, women would need to provide a spit sample so that their DNA could be analysed.

- How would you feel about providing a spit sample?

Probe: What are your reasons for feeling that way?

- What could stop women providing a spit sample?

Probe: How could these barriers be minimised?

- What could be done to help women provide a spit sample?

Prompt: What would be your preferred route (online, telephone etc) for accessing help?

1. To find out risk, women would need to have a mammogram (breast x-ray). This will allow assessment of the different types of tissue in the breast which is known to be related to breast cancer risk.

- How would you feel about having a breast x-ray?

Probe: What are your reasons for feeling that way?

- What could stop women attending a breast x-ray?

Probe: How could these barriers be minimised?

- What could be done to help women attend a breast x-ray?

Prompt: What would be your preferred route (online, telephone etc.) for accessing help? How could non-attenders be encouraged to attend?

1. Depending on answers to the questionnaire, completing a breast cancer risk assessment will not be the right option for every woman. In these cases, women will be directed to another healthcare pathway.

- How would you feel if you were invited but later found out you could not complete a risk assessment?

Probe: What are your reasons for feeling that way?

- What could be done to help women in this situation?

**Communication of risk**

1. Results from the questionnaire, spit sample and breast x-ray will be combined to give a risk score and category.

- How would you like to receive your risk assessment results?
- How do you think risk information should be presented?

Prompts: numerically, graphically, labelling of categories

- What information would you like in the risk feedback?

Prompt: would the information you like differ according to level of risk received e.g. low vs high?

- If you had any queries about your results, how and with whom would you like to discuss this with?
- What information about your breast cancer risk do you think would be important for your GP to know?

1. Women identified at increased risk of developing breast cancer would be invited to attend an appointment at a risk and prevention clinic. During this appointment, care pathways to reduce breast cancer risk such as earlier access to breast screening and medication to prevent cancer from developing will be discussed.

- How would you feel about this?

Probe: What are your reasons for feeling that way?

- How would you feel about using breast cancer risk to determine access to healthcare pathways?

Probe: What are your reasons for feeling that way?

What are your thoughts about risk management strategies such as chemoprevention or lifestyle changes?

Probe: What are your reasons for feeling that way? Do you believe in prevention?

- What could stop women attending an appointment at the risk and prevention clinic?

Prompt: How could these barriers be minimised?

- What could be done to help women attend the appointment?

Prompt: What would be your preferred route (online, telephone etc.) for accessing help?

1. Can you think of any additional issues which are relevant for women as they turn 30 which we should consider when designing this breast cancer risk assessment pathway/that might impact engagement with a breast cancer risk assessment programme?

**Finishing comments**

Thanks for your time today. We really do appreciate it.

- Is there anything else you want to add?
- Is there anything you thought you would talk about today that you haven’t had a chance to say and want to mention?
- Do you have any questions for me?
